# Selective suppression of rapid eye movement sleep increases next-day negative affect and amygdala responses to social exclusion

**DOI:** 10.1038/s41598-020-74169-8

**Published:** 2020-10-14

**Authors:** Robert W. Glosemeyer, Susanne Diekelmann, Werner Cassel, Karl Kesper, Ulrich Koehler, Stefan Westermann, Armin Steffen, Stefan Borgwardt, Ines Wilhelm, Laura Müller-Pinzler, Frieder M. Paulus, Sören Krach, David S. Stolz

**Affiliations:** 1 Social Neuroscience Lab, Department of Psychiatry and Psychotherapy, University of Lübeck, Ratzeburger Allee 160, 23538 Lübeck, Germany; 2 Translational Psychiatry Unit (TPU), Department of Psychiatry and Psychotherapy, University of Lübeck, Ratzeburger Allee 160, 23538 Lübeck, Germany; 3 Institute of Medical Psychology and Behavioral Neurobiology, University Tübingen, Otfried-Müller-Str. 25, 72076 Tübingen, Germany; 4 Department of Psychiatry and Psychotherapy, University Hospital Tübingen, Calwerstr. 14, 72074 Tübingen, Germany; 5 Division of Pneumology, Intensive Care and Sleep Medicine, Department of Internal Medicine, Hospital of the University of Marburg, Marburg, Germany; 6 Medical School Hamburg, Hamburg, Germany; 7 Department of Otorhinolaryngology, University of Lübeck, Ratzeburger Allee 160, 23538 Lübeck, Germany

**Keywords:** Circadian rhythms and sleep, Emotion, Social neuroscience

## Abstract

Healthy sleep, positive general affect, and the ability to regulate emotional experiences are fundamental for well-being. In contrast, various mental disorders are associated with altered rapid eye movement (REM) sleep, negative affect, and diminished emotion regulation abilities. However, the neural processes mediating the relationship between these different phenomena are still not fully understood. In the present study of 42 healthy volunteers, we investigated the effects of selective REM sleep suppression (REMS) on general affect, as well as on feelings of social exclusion, cognitive reappraisal (CRA) of emotions, and their neural underpinnings. Using functional magnetic resonance imaging we show that, on the morning following sleep suppression, REMS increases general negative affect, enhances amygdala responses and alters its functional connectivity with anterior cingulate cortex during passively experienced experimental social exclusion. However, we did not find effects of REMS on subjective emotional ratings in response to social exclusion, their regulation using CRA, nor on functional amygdala connectivity while participants employed CRA. Our study supports the notion that REM sleep is important for affective processes, but emphasizes the need for future research to systematically investigate how REMS impacts different domains of affective experience and their neural correlates, in both healthy and (sub-)clinical populations.

## Introduction

Sleep is fundamental for general well-being^1,2^. Healthy sleep consists of two repeatedly cycling types of sleep: non-rapid eye movement sleep (NREM) including slow-wave sleep (SWS) phases of deep sleep and rapid eye movement sleep (REM). Over the course of the night, the percentage of SWS decreases while REM sleep percentage increases towards the morning^3^. In many occasions however, sleep is fragmented or shortened (e.g. due to stress, early school or work starts and late bedtimes) and even minor sleep deprivation can have broad, short-term as well as long-term consequences for health and well-being. Sleep disturbances affect health on the level of immune regulation^4–6^ and metabolic markers^7,8^. Furthermore, sleep loss negatively impacts cognitive processing and emotional reactivity^9,10^, as well as social behavior, leading to withdrawal from social interaction and feelings of loneliness^11^.

Interestingly, most mental disorders are associated with sleep peculiarities. Insomnia is comorbid in 85–90% of individuals diagnosed with major depression^12–14^. Alterations in REM sleep architecture (REM sleep latency, density and distribution) are particularly prominent in most mental disorders such as major depression, bipolar disorder and Posttraumatic Stress Disorder (PTSD)^15,16^. Given that these disorders are mainly characterized by altered affective experience during daytime, it is interesting to note that inadequate sleep itself has been shown to impact next day emotionality^17^. Additionally, sleep loss can impede emotion regulation capacities^18^, likely contributing further to emotional detriments. Coherently, recent studies even suggest sleep abnormalities as being causal agents in mood disorders rather than just a symptom^13,19^.

Although sleep disturbances are a hallmark of mood disorders, the specific roles of different sleep stages in supporting cognitive and emotional functioning are not well understood. Increasing evidence suggests that SWS and associated neurophysiological processes support the consolidation of newly encoded declarative memories^20,21^. REM sleep, on the other hand, is particularly important for emotional processing, including emotional reactivity^22,23^ and the formation of emotional memories^24,25^. REM-sleep dreaming was also found to attenuate residual emotional load from the day before^26,27^. However, only very few studies have examined the effects of experimentally induced, selective suppression of REM sleep during an otherwise normal night of sleep. The existing literature suggests that the selective deprivation of REM sleep mainly disturbs the consolidation of emotional memories^28–30^, whereas the selective suppression of SWS mainly impairs emotionally-neutral declarative memory encoding and consolidation^31,32^. These experimental findings are in line with clinical observations suggesting that REM sleep in particular is closely tied with emotional functioning^19^.

In order to better understand the processes underlying the interaction of sleep and emotion, research increasingly addresses the neural mechanisms associated with REM sleep-dependent emotional functioning. REM sleep presumably serves to reorganize neural representations of emotional experiences by distributed reactivation of these representations in amygdala, hippocampus, and neocortical structures, driven by synchronized theta oscillations between these regions and increased cholinergic activity. Together with reduced adrenergic neurotransmission during REM sleep, these processes allow for concomitant reductions of the affective arousal associated with the experiences^19,33^. Coherently, diminished central adrenergic activity, measured by frontal gamma activity, has been shown to predict behavioral and amygdala adaptation to emotional stimuli presented once before and once after sleep^34^. The amygdala, a cluster of nuclei in the temporal lobes and parts of the limbic system^35^, and the limbic system more generally, are widely associated with the processing of affectively laden stimuli^36^ and play an important role in the guidance of behavioral responses to such stimuli^37,38^. Recent findings show that fragmented REM sleep, presumably caused by recurring, noradrenergic activity, diminishes amygdala adaptation to repeated emotional stimulation^39^. Under conditions of total sleep deprivation, next-day negative affect is accompanied by increased amygdala reactivity and decreased functional coupling of the medial prefrontal cortex (MPFC) with limbic structures^40^. Since the MPFC is thought to exert inhibitory control on the amygdala^41^, this finding has been interpreted to reflect a failure of top-down control in the regulation of appropriate emotional responsivity^40^. In addition, Ben Simon and colleagues directly examined the effect of sleep deprivation on the neural correlates of next day emotion reactivity. Following sleep deprivation, there was no indication for valence-specific processing of affective pictures in the amygdala. However, following a night of sleep, a low amount of REM sleep was associated with a decline in anterior cingulate cortex (ACC)-amygdala connectivity^18^, possibly reflecting a specific effect of REM sleep on cognitive control of emotions^42^.

Successful cognitive control of emotions is regarded to be an essential prerequisite of mental health^43,44^. In daily life, emotions are constantly regulated either implicitly or explicitly by applying specific cognitive strategies like suppression (e.g. distracting the attention away from unpleasant emotional experiences) or reappraisal (e.g. reinterpreting an unpleasant emotional situation). Emotion regulation thus refers to the ability to “influence which emotions we have, when we have them, and how we experience and express these emotions” (p.497^45^). Interestingly, correlational evidence indicates that the success of emotion regulation is associated with sleep quality^46^. In this study, participants were asked to engage in cognitive reappraisal (CRA)^47^, in this case applying a previously learned cognitive strategy to “redirect the spontaneous flow of emotions” (p. 6^48^), while watching a sadness-inducing film. The ability to decrease self-reported sadness using CRA compared to baseline was lower in participants who reported poorer sleep quality during the preceding week^46^.

Despite their substantial clinical significance, the neural mechanisms of the effect of REM sleep on the efficacy of regulation strategies in ameliorating unpleasant affect remain to be understood in more detail. This lack of evidence holds especially for studies employing socially immersive paradigms^49^, which is surprising considering the relevance of social interaction for mental health^50^. To fill this gap, we simulated social exclusion in a laboratory setting using the so-called *Cyberball*^51^. *Cyberball* is a virtual ball-tossing paradigm where participants are playing with a preset computer program while believing that they are playing with two other human participants. By manipulating the number of ball-tosses towards the participant, the degree of social inclusion can be controlled experimentally. A recent meta-analysis of neuroimaging studies using the *Cyberball* game found reliably greater activations in cingulate and prefrontal regions when participants were excluded compared to when they were included^52^. Furthermore, evidence suggests that the distressing experience of social exclusion might share neural architecture with the affective processing of physical pain^53–56^. Although behavioral consequences and neural activation patterns associated with social exclusion have been studied quite intensively, there is scarce research on intervening cognitive appraisals and the role that sleep plays with regard to feelings of social exclusion^57^. To our knowledge, only one study investigated the impact of total sleep deprivation on feelings of social exclusion but could not find any specific effect of sleep. Furthermore, this study neither focused on specific sleep stages nor did it address the underlying neural mechanisms^58^. Hence, the present study aims to extend this field of research by combining the *Cyberball* with functional magnetic resonance imaging (fMRI), suppression of specific sleep stages, and a manipulation of employing cognitive reappraisal strategies.

The aim of the present study was to examine how selective REM sleep suppression impacts the following day general affect, emotional reactivity, and associated neural mechanisms of emotion regulation during the acute experience of social exclusion. In a between-subjects design we invited participants to a combined polysomnography and fMRI study. After a first night allowing regular sleep (henceforth: habituation night), for the second night (experimental night) participants were randomly allocated to either a REM sleep suppression (REMS) group or one of two control groups: a non-suppression control group with regular sleep (CTL) or a group with similar amounts of awakenings, but where suppression targeted phases of slow wave sleep (SWSS). To assess the impact of REMS on general affect, subjects filled in the Positive and Negative Affect Schedule (PANAS)^59^ right before going to bed and after waking up on both nights. On the morning after the experimental night, subjects participated in the *Cyberball* during fMRI scanning to induce feelings of social exclusion. All participants engaged in two sessions of the game. In the first session, participants played the game without any instructions. In the second session, participants were instructed to actively regulate their emotions by applying the previously learned CRA.

We hypothesized that selective REMS (vs. SWSS and regular sleep) generally reduces positive and increases negative affect. Furthermore, we expected that selective REMS (vs. SWSS and regular sleep) increases emotional reactivity during social exclusion and dampens the effect of CRA on emotional reactivity. On the level of neural systems, we explored whether REMS leads to altered functional activity and connectivity of (para-)limbic areas during social exclusion and their modulation by cognitive reappraisal. Precisely, we focused on the bilateral amygdala, ACC, insula, and hippocampus, as these regions are broadly implicated in emotional processing^35,36^, and more specifically in sleep-dependent emotional adaptation^19,40^ as well as regulation of emotions^17^.

## Results

### Experimental sleep manipulation selectively reduces REM sleep percentage

**Figure 1:**
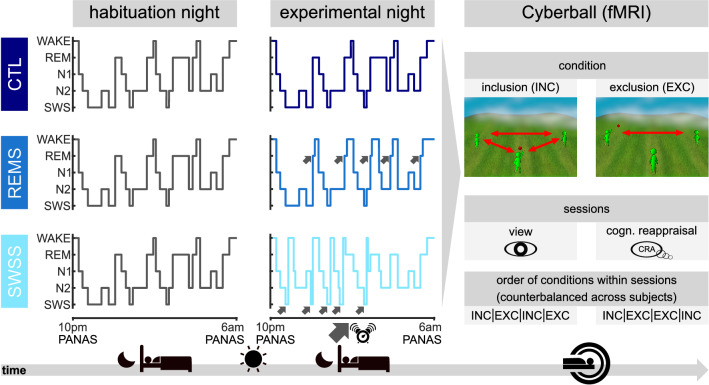
Summary of the experimental procedure. Three groups of subjects spent two consecutive nights in the sleep laboratory. During both nights, polysomnography was recorded. In the second night, two groups were woken up as soon as they entered REM sleep or SWS (groups REMS and SWSS, respectively), while the control group was not woken up (CTL). The grey arrows indicate onsets of REM sleep and SWS upon which the respective groups were awakened. After waking up on the second morning, all subjects performed two sessions of the *Cyberball* game while inside the fMRI scanner^91^. The game included a total of eight blocks, with four inclusion (INC) and four exclusion (EXC) blocks. During inclusion, subjects received the ball continuously throughout the block, while during exclusion, the other two players stopped throwing the ball to the participant soon after the beginning of the block, effectively excluding the participant from the game. Additionally, during the first four blocks (half INC, half EXC), subjects simply performed the task (VIEW), while during the second four blocks (half INC, half EXC) they were instructed to use cognitive reappraisal (CRA) to regulate their emotions.

**Figure 2:**
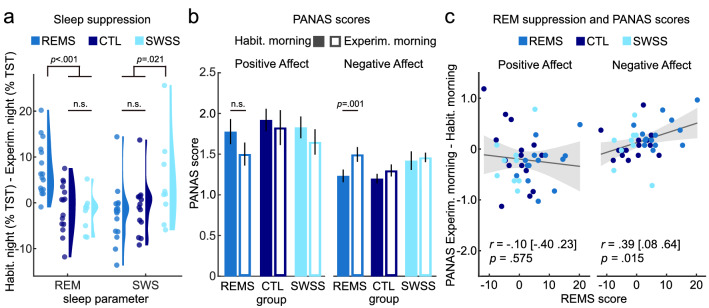
Selective sleep suppression and general affect. (**a**) REM sleep was significantly more reduced in the REMS group than in the other two groups, whereas SWS suppression was significantly stronger in the SWSS group than in the other two groups. Suppression scores for REM (SWS) sleep are the difference of percentage REM (SWS) of TST between the habituation night minus the experimental night. (**b**) Ratings of general positive and negative affect, measured with the Positive And Negative Affect Scale (PANAS)^59^ on the mornings after the habituation night and after the experimental night, separately for the three groups. (**c**) Changes in negative (right) but not positive affect (left) from the habituation night to the experimental night correlate significantly with REM sleep suppression scores across all groups. Shaded areas represent 95% confidence intervals for simple bivariate regression. Error bars represent standard error of the mean. *n.s. *not significant.

The experimental sleep manipulation (see Fig. 1) successfully suppressed REM sleep in the REMS group during the experimental night (REMS score = %REM sleep in habituation − %REM sleep in experimental night; effect of group: *F*(2,37) = 13.21, *p* < 0.001, *η*^2^ = 0.42; planned contrast REMS > others: *t*(38) = 5.17, one-sided *p* < 0.001). The REMS and SWSS groups were experimentally disturbed similarly often in the experimental night (REMS: *M* = 15.29, *SD* = 5.25; SWSS: *M* = 15.70, *SD* = 8.71; *t*(25) = 0.15, *p* = 0.881), and the SWSS and CTL groups were similar with regard to the amount of REM sleep suppression (*t*(22) = 0.52, two-sided *p* = 0.608; see Table 1 and Fig. 2a for details).

**Table 1:** Sleep measures.

	Group	Habituation night		Experimental night		Habituation vs. experimental night		
		*M*	*SD*	*M*	*SD*	*t*	*df*	*p*
TST (min)	CTL	369.94	78.06	414.59	26.08	− 2.79	14	0.015
	REMS	410.86	40.24	399.05	39.02	1.13	15	0.277
	SWSS	384.63	54.15	402.96	31.28	− 1.41	8	0.197
N1 (% TST)	CTL	1.87	2.76	0.58	0.96	1.80	14	0.093
	REMS	0.60	0.65	1.72	2.54	− 1.78	15	0.096
	SWSS	2.47	2.57	1.18	1.39	1.36	8	0.212
N2 (% TST)	CTL	61.82	7.27	61.11	8.53	0.32	14	0.751
	REMS	64.83	4.69	68.66	7.62	− 2.12	15	0.051
	SWSS	65.68	8.44	69.33	8.29	− 1.37	8	0.209
SWS (% TST)	CTL	17.11	6.21	18.04	7.92	− 0.65	14	0.525
	REMS	14.18	6.88	16.86	5.62	− 1.60	15	0.131
	SWSS	13.98	7.43	9.83	4.82	1.40	8	0.199
REM (% TST)	CTL	19.21	6.33	20.27	4.39	− 0.77	14	0.455
	REMS	20.39	5.86	12.76	6.16	5.00	15	< 0.001
	SWSS	17.42	6.56	19.65	8.87	− 1.60	8	0.148
WASO (min)	CTL	67.16	50.46	51.06	29.00	1.34	14	0.201
	REMS	47.01	40.18	71.10	42.66	− 2.47	15	0.026
	SWSS	57.57	38.50	61.63	37.96	0.08	8	0.941
WASO/(TST + WASO)	CTL	0.16	0.13	0.11	0.06	1.79	14	0.096
	REMS	0.10	0.09	0.15	0.09	− 2.32	15	0.035
	SWSS	0.13	0.10	0.13	0.08	0.36	8	0.728
REM latency (min)	CTL	142.97	68.32	106.10	51.71	1.78	14	0.097
	REMS	101.19	43.15	154.12	93.11	− 1.77	15	0.096
	SWSS	137.78	86.27	123.35	82.01	1.44	8	0.189
Wake phases	CTL	8.13	3.09	9.00	4.21	− 1.11	14	0.285
	REMS	7.75	3.66	11.47	4.90	− 2.50	15	0.024
	SWSS	11.11	6.64	15.00	6.68	− 1.08	8	0.311
Sleep efficiency	CTL	77.19	16.72	83.32	8.17	− 1.82	14	0.091
	REMS	84.71	8.62	79.91	8.81	1.93	15	0.072
	SWSS	80.93	12.68	78.66	8.38	0.14	8	0.891

In both the REMS group and the SWSS group, PSG data quality from one subject was not suitable for analysis in the habituation night.

*CTL *control group with undisturbed sleep, *REMS *rapid eye movement sleep suppression group, *SWSS *slow wave sleep suppression group, *TST *total sleep time (time from sleep onset to morning awakening after the time awake during the night is subtracted), *N1 *sleep stage 1, *N2 *sleep stage 2, *SWS *sleep stage N3, *REM *rapid eye movement sleep, *WASO *wakefulness after sleep onset to morning awakening, *M *mean, *SD *standard deviation. *p*-values are uncorrected.

### REM sleep predicts general negative affect

General affect ratings in the PANAS obtained in the morning differed significantly between habituation and experimental night (effect of night: positive affect (PA): *F*(1,37) = 4.55, *p* = 0.040, $\eta_{p}^{2}$ = 0.11; negative affect (NA): *F*(1,37) = 9.32, *p* = 0.004, $\eta_{p}^{2}$ = 0.20), while type of suppression had no significant effect (PA: *F*(2,37) = 0.74, *p* = 0.484, $\eta_{p}^{2}$ = 0.04; NA: *F*(2,37) = 2.21, *p* = 0.124, $\eta_{p}^{2}$ = 0.11). The interaction effect was not significant for positive affect (PA: *F*(2,37) = 0.34, *p* = 0.713, $\eta_{p}^{2}$ = 0.02) but approached significance for negative affect (NA: *F*(2,37) = 3.08, *p* = 0.058, $\eta_{p}^{2}$ = 0.14). Across groups, post-hoc comparisons demonstrated that positive affect was lower and negative affect was higher after the experimental night as compared to the habituation night (PA: *t*(39) = 2.20, two-sided *p* = 0.034, *d* = 0.35, 95% CI = [0.03; 0.67]; NA: *t*(39) = − 3.23, two-sided *p* = 0.003, Cohen’s *d* = − 0.51, 95% CI = [− 0.84; − 0.18]), with the increase in negative affect being most pronounced in the REMS group (*t*(14) = 4.47, *p* = 0.001, Cohen’s *d* = 1.15, 95% CI = [0.48; 1.80]; see Fig. 2b). A planned contrast showed that this increase in negative affect was stronger in the REMS group as compared to the other groups (*t*(38) = 2.47, two-sided *p* = 0.037, Cohen’s *d* = 0.81, 95% CI = [0.14; 1.47]), whereas no group difference could be observed for change in positive affect (*t*(38) = − 0.72, two-sided *p* = 0.950, Cohen’s *d* = 0.24, 95% CI = [− 0.88; 0.41], all *p*-values Bonferroni-corrected).

Regarding the evening ratings, type of sleep suppression did not have any significant effects or interactions, neither for PA nor for NA (all *p*s > 0.110; see Supplementary Tables S2 and S3 for details). Since low quality sleep, and particularly REM sleep, can interfere with emotional dissipation overnight^39^, we tested whether type of sleep suppression influenced the change in NA or PA from the evening before to the morning after the experimental night, which was not the case (all *p*-values > 0.268; see Supplementary Tables S5 and S6). Across groups, however, increases in morning negative affect from habituation to experimental night could be predicted by the REMS score (%REM sleep in habituation − %REM sleep in experimental night; Pearson’s *r* = 0.39, one-sided *p* = 0.015, 95% CI = [0.08; 0.63]; Fig. 2c), even after controlling for changes in SWS, total sleep time (TST) and wakefulness after sleep onset in a multiple linear regression model (see Table 2 and “Methods” for details on PSG recordings and analysis, which all followed standard American Academy Of Sleep Medicine (AASM) guidelines^60^).

**Table 2:** Regression of change in negative affect (∆ Experimental morning − habituation morning) on sleep deprivation parameters.

	*B*	*SE*	*β*	*t*	*p*	sign
(Intercept)	0.098	0.060		1.64	0.110	n.s.
REM sleep suppression	0.020	0.008	0.406	2.60	0.014	*
SWS sleep suppression	− 0.000	0.008	− 0.002	− 0.01	0.989	n.s.
TST suppression	0.002	0.002	0.404	1.60	0.118	n.s.
WASO increase	0.005	0.002	0.638	2.95	0.006	**

*REM *rapid eye movement sleep, *SWS *slow wave sleep, *TST *total sleep time, *WASO *wakefulness after sleep onset, *n.s.* not significant.

* *p* < 0.05; **p < 0.01.

### No effect of selective REM sleep suppression on self-reported emotional responses to social exclusion

By means of the *Cyberball*, we successfully induced the unpleasant feeling of social exclusion (main effect of INC/EXC: *F*(1,39) = 131.65, *p* < 0.001, $\eta_{p}^{2}$ = 0.771; see Table 3 for descriptive statistics), thereby replicating earlier findings^56^. Moreover, if participants were asked to engage in emotion regulation using cognitive reappraisal, feelings of being excluded could be toned down significantly (main effect of VIEW/CRA: *F*(1,39) = 66.99, *p* < 0.001, $\eta_{p}^{2}$ = 0.632). This effect was most strongly pronounced during trials of social exclusion (interaction effect INC/EXC × VIEW/CRA: *F*(1,39) = 44.97, *p* < 0.001, $\eta_{p}^{2}$ = 0.536), as verified using post-hoc t-tests (VIEW > CRA: EXC: *t*(41) = 8.36, one-sided *p* < 0.001, *d* = 1.29, 95% CI = [0.87; 1.70]; INC: *t*(41) = 3.62, one-sided *p* < 0.001, *d* = 0.56, 95% CI = [0.23; 0.88]; difference between EXC and INC for VIEW minus CRA: *t*(41) = 7.01, two-sided *p* < 0.001, *d* = 1.08, 95% CI = [0.70; 1.46]).

**Table 3:** Feelings of being excluded, separately for groups and conditions.

Group	INC/VIEW		EXC/VIEW					INC/CRA					EXC/CRA				
	*M*	*SD*	*M*	*SD*	*t*	*df*	*p*	*M*	*SD*	*t*	*df*	*p*	*M*	*SD*	*t*	*df*	*p*
CTL	2.20	1.25	6.67	1.92	− 10.68	14	< 0.001	1.50	0.78	2.59	14	0.022	3.67	2.50	− 2.07	14	0.058
REMS	2.00	1.21	6.38	2.90	− 7.29	16	< 0.001	1.62	0.86	1.77	16	0.059	2.82	2.33	− 2.04	16	0.097
SWSS	2.25	0.98	5.90	2.49	− 5.41	9	< 0.001	1.75	1.46	1.79	9	0.107	2.60	1.51	− 1.08	9	0.310

Inferential statistics report results from paired *t* tests of INC/VIEW against each remaining condition. *p*-values are uncorrected.

*EXC *exclusion condition, *INC *inclusion condition, *VIEW *passive viewing session, *CRA *cognitive reappraisal session, *CTL *control group, *REMS *REM sleep suppression group, *SWSS *slow wave sleep suppression group.

However, regarding our second hypothesis, type of sleep suppression neither had a significant impact on emotions after social exclusion (INC/EXC × group interaction: *F*(2,39) = 1.47, *p* = 0.243, $\eta_{p}^{2}$ = 0.070) nor on the effect of emotion regulation (VIEW/CRA × group interaction: *F*(2,39) = 0.027, *p* = 0.973, $\eta_{p}^{2}$ = 0.001). Last, we did not find a statistically significant three-way interaction on the self-reported emotions after social exclusion (INC/EXC × VIEW/CRA × group interaction: *F*(2,39) = 0.45, *p* = 0.639, $\eta_{p}^{2}$ = 0.023). Similarly, no significant main effect of or interactions with type of sleep suppression were found when contrasting REMS against the other two groups (all *p*-values > 0.41).

### REM sleep suppression alters amygdala activity during social exclusion

Similar to earlier studies, we found that during social exclusion participants’ neural activity in the left and right hippocampus (left: x, y, z (mm): − 36, − 40, − 6; *T* = 7.12, *k* = 88, *p* < 0.001, FWE-corrected; right: 36, − 32, − 8; *T* = 4.86, *k* = 3, *p* = 0.023, all *p*-values refer to FWE-corrected tests at peak-level) as well as the right insula (34, − 10, 22; *T* = 5.03, *k* = 12, *p* = 0.015, FWE-corrected) was significantly increased compared to the inclusion condition^61,62^. In addition, across groups, neural activity in the right anterior insula (32, 28, − 4; *T* = 4.97, *k* = 11, *p* = 0.017) and the dorsal ACC (2, 14, 22; *T* = 4.69, *k* = 3, *p* = 0.034, FWE-corrected) was significantly increased during VIEW as compared to CRA blocks. Testing whether these two main effects interacted or whether they were modulated by type of sleep suppression did not yield any significant effects. This held both when testing for differences between any of the three experimental groups and when comparing the REMS group against the other two groups combined.

**Figure 3:**
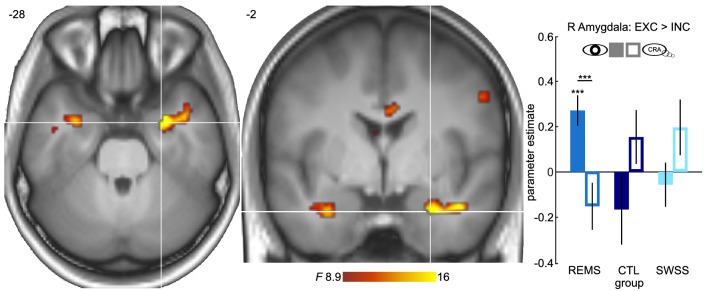
Neural responses to ostracism are altered by selective REM sleep suppression. **Left** Neural responses for the contrast [EXC/VIEW > INC/VIEW] > [EXC/CRA > INC/CRA] differed significantly between the REMS group and the other two groups in the right amygdala, (peak MNI-coordinates: x = 26, y = − 2, z = − 28, surviving FWE-correction at *p* < 0.05 inside the a priori mask). Displayed at *p* < 0.005, uncorrected, for visualization purposes. **Right** Parameter estimates for the contrast EXC > INC in the VIEW session were positive in the REMS group, but did not deviate from 0 in the other two groups, nor in the CRA session for any of the three groups. In addition, parameter estimates for EXC > INC were significantly more positive in the REMS group during VIEW than during CRA, while sessions did not differ in the other two groups. See main text for details. ***p < 0.001. Error bars represent standard error of the mean.

Next, we tested the three-way interaction of EXC/INC, VIEW/CRA, and type of sleep suppression. A test of differences between any of the three groups for the two-way interaction contrast [EXC/VIEW > INC/VIEW] > [EXC/CRA > INC/CRA] was not significant. However, comparing the REMS group to the other two groups indicated differential neural responses in the right amygdala for the REMS group (28, 0, − 30; *F* = 27.84, *k* = 5, *p* = 0.016, FWE-corrected; see Fig. 3). Precisely, the contrast EXC > INC was positive during VIEW in the REMS group, but did not differ from zero in the other groups (REMS: *t*(16) = 3.34, two-sided *p* = 0.025; CTL: *t*(14) = − 0.87, two-sided *p* = 1.000; SWSS: *t*(9) = − 0.66, two-sided *p* = 1.000). In addition, EXC > INC was more positive during VIEW than during CRA in the REMS group (*t*(16) = 4.77, two-sided *p* < 0.001), but not in the other groups (CTL: *t*(14) = − 2.15, two-sided *p* = 0.149; SWSS: *t*(9) = − 2.36, two-sided *p* = 0.128; all p-values Bonferroni-corrected). This pattern of results suggests that REM-sleep suppression increases amygdala signaling of information that is relevant to the individual’s social well-being, particularly when not engaging in CRA.

### REM sleep suppression alters amygdala–ACC connectivity independently of CRA

Since amygdala activity for EXC > INC differed between the REMS group and the other two groups during VIEW but not during CRA, we tested whether the amygdala would show a specific pattern of functional connectivity which could put these findings into a broader perspective. Using psychophysiological interaction (PPI) analyses, we tested whether functional connectivity of the amygdala differed between EXC and INC blocks (see “Methods”), and whether this depended on session, group, or their interaction.

For the right amygdala, we did not find any functional connectivity differences between exclusion and inclusion across groups in any of the sessions. However, during the VIEW condition where participants simply participated in the *Cyberball* without engaging in cognitive reappraisal, functional connectivity between the right amygdala and right ACC differed between the REMS group and the other two groups (14, 40, 28, *F* = 22.21, *k* = 2, *p* = 0.029, FWE-corrected in the a priori mask). Precisely, functional connectivity between these regions was elevated in the REMS group during blocks in which participants were excluded as compared to blocks in which they were included (parameter estimates extracted from ACC peak: *t*(16) = 3.90, *p* = 0.001, *d* = 0.95, 95% CI = [0.36; 1.51]). This increased amygdala–ACC functional connectivity was not evident in the other two groups (CTL: *t*(14) = − 1.84, *p* = 1.00, *d* = − 0.48, 95% CI = [− 1.00; 0.07]; SWSS: *t*(9) = − 0.83, *p* = 1.00, *d* = − 0.26, 95% CI = [− 0.89; 0.36]; Bonferroni-corrected, two-sided *p*-values). Thus, during passively experienced social exclusion, selective REM sleep suppression may entail relatively more positive connectivity (or less inhibitory connectivity^42^) between ACC and amygdala as compared to social inclusion. During CRA, selective REM sleep suppression did not significantly alter functional connectivity in any region within the a priori mask. However, a direct comparison between sessions of the effect in right ACC was not significant in any of the three groups (REMS: *t*(16) = 2.56, *p* = 0.063, *d* = 0.62, 95% CI = [0.10; 1.13]; CTL: *t*(14) = − 2.18, *p* = 0.15, *d* = − 0.56, 95% CI = [− 1.10; − 0.01]; SWSS: *t*(9) = 0.29, *p* = 1.00, *d* = 0.09, 95% CI = [− 0.53; 0.71]; Bonferroni-corrected, two-sided *p*-values). Similarly, no other brain region showed a significant group by session interaction, and no significant effects were found when performing the same analyses for the left amygdala. In sum, selective REM sleep suppression increased functional connectivity between the right amygdala and right ACC when social exclusion was passively experienced, but there was no evidence for specificity of this effect when compared to cognitive reappraisal during social exclusion.

## Discussion

Nearly everyone can relate to the devastating effects of a sleepless or interrupted night on one’s next day mood. While the effect of total sleep deprivation on emotional reactivity has been investigated intensively in the past^63,64^ the present study focused on the specific impact of selective REM sleep suppression (REMS) on general affect, as well as emotion regulation and its neural correlates under conditions of social exclusion. We found that lower amounts of REM sleep across all participants were associated with higher levels of general negative affect in the next morning, a finding that is in line with previous literature implicating REM sleep in affective functioning^65^. Despite this general effect, however, our findings do not provide evidence for a direct link of REMS with the subjective emotional response to experimentally induced social exclusion. The ability to regulate one’s negative emotions during social exclusion was also not affected by prior REMS, which was an unexpected finding. Interestingly though, despite no changes in subjectively reported emotions, neural activity and connectivity of the amygdala were altered after REMS when participants passively experienced social exclusion.

The disparate effects of REMS on general affect and specific emotional responses to social exclusion indicate that selective sleep deprivation might differentially impact specific domains of affective processing. That is, results may vary between general state-like morning affect (e.g. as measured using the PANAS)^66^, the processing and responding to affective material (e.g. facial emotion recognition^67^) or on the direct induction of emotional states (e.g. inducing the unpleasant experience of social exclusion^58^, pain processing^66^). For instance, general unspecific affect may function differently than emotional reactions to specific elicitors^68^, potentially moderating the effect of sleep deprivation or REMS in particular on these different affective processes^69^. However, at least one study found that general affect was not influenced by REMS, which contradicts our findings^30^. In addition, one previous study did not provide evidence for any detrimental effect of total sleep deprivation on feelings of social exclusion^58^, and apart from the present study there is no further work that directly examined the effect of selective REMS on experimentally induced emotional states. Last, we are not aware of any study systematically comparing the extent to which the different psychological aspects of affective experience are susceptible to selective suppression of sleep stages. Taken together, the specific interaction of sleep and specifically REM sleep with general affect, in contrast to more confined emotional responses, demands more in-depths analyses of different kinds of experimental designs and dependent variables^69^.

On the neural level, selective REMS was associated with increased activity in the right amygdala when social exclusion was passively endured as compared to situations where an active regulation of affect was requested. The specificity of this effect for REMS highlights the importance of this sleep stage for emotional functioning^22–25^. Further, this finding nicely connects previous studies that showed amygdala responses to viewing negative emotional stimuli increased after total sleep deprivation^40^, depended on intact REM sleep in particular^39,70^, and correlated with autonomic responses to psychosocial stress^71^. The amygdala is strongly associated with emotional processing^35,72^ and has anatomical connections to the anterior insula and the ACC^73,74^. As part of this so-called salience network^75^, the amygdala’s assumed function of signaling the relevance of information is central for the domain of affective experiences^72,76^. It is assumed that during REM sleep, neural representations of previously experienced emotional events are reorganized and lose their affective tone, while adrenergic signaling is disengaged^19,33^. Our findings extend this perspective in that insufficient REM sleep does not only inhibit adaptation to previous emotional experiences during the night^39^, but also leads to altered amygdala responses to newly experienced social situations on the next day. This increase in amygdala signaling was paralleled by relatively more positive connectivity with anterior cingulate cortex, which could indicate that REMS diminishes amygdala inhibition by the ACC^42^ when aversive social situations are passively endured. Interestingly, we did not find evidence that REMS interferes with the ability to apply cognitive reappraisal, as indicated by the ratings of subjectively felt social exclusion. Additionally, in contrast to passively experienced social exclusion, no effect of REMS on amygdala connectivity was observed during cognitive reappraisal. Together, this may indicate that the ability to deliberately counteract the impact of social exclusion was not impaired in REM sleep deprived subjects. However, our data remain inconclusive regarding the question of whether REMS differentially impacts amygdala connectivity depending on whether or not emotions are actively regulated, hence calling for further research.

We can only speculate why the REMS–induced alterations in brain function during passively experienced social exclusion did not manifest on the level of increased subjective emotional reactivity. Previous research provides several explanations that might be helpful to understand these disparate findings. First, the effects of experimental short-term sleep manipulations might be strongest immediately after awakening and may be readily washed out thereafter^77^, and may thus not have lasted until the fMRI session in the present study. This may hold in particular for selective suppression of specific sleep stages rather than total sleep deprivation, which produces stronger and more long-lasting effects^77^. The efficacy of REMS in the present study may have been too limited to surface on the level of emotion ratings later in the morning. In contrast, the altered brain activity in the amygdala could indicate that even small changes in REM sleep can influence brain systems implicated in regulating responses to affectively salient stimuli^35,72^, while they are too small to penetrate the level of subjective experiences, which are reportedly processed further downstream^71,78^. Yet, what speaks against this explanation are findings by Wiesner and colleagues^30^ or Morgenthaler and colleagues^79^ who applied even more rigorous REM sleep deprivation, achieving a mean REM sleep percentage of around one percent of TST, but nevertheless could not find REM sleep related behavioral effects during emotion recognition tasks. Similarly, Liu and colleagues compared the effect of a 24 h sleep deprivation to regular sleep on the experience of distress following social exclusion in the *Cyberball* game^58^. As in the present study, the authors did not find an effect of sleep deprivation on the subjective experience of social rejection.

Regardless of the timing and potential washout during the day, the lack of significant findings for the experience of social exclusion might also relate to the fact that emotional experiences are assumed to arise through more complex processes than neural activity in single regions. Precisely, current emotion theory posits that emotional experiences can be understood in terms of situated conceptualizations, that is, ongoing interpretations of interoceptive states through application of socially agreed upon emotion concepts (such as *fear*)^80,81^. Thus, while the amygdala showed increased reactivity towards potentially threatening conditions, signaling greater homeostatic imbalance, the distinct assessment of emotional responses to ostracism, such as feelings of exclusion, might touch a different facet of affective construal. That is, the affective salience of information tracked by amygdala activity^72,76^ may be modulated by REMS. However, the construal of emotional experience is assumed not to rely on activity in single regions, but on the dynamic interaction of various neural systems supporting multiple psychological processes of emotional experience apart from affective salience^80,81^. Hence, amygdala responses to psychosocial stress may not necessarily influence the construal of subjective emotion ratings, that presumably depend on additional networks involving prefrontal cortical regions, as suggested by previous studies^17,71,82^.

The unexpected disparity between findings on the neural level and the subjective emotion ratings may also relate to the possibility that healthy participants have resources to compensate neural signaling of adverse experiences. It has been shown that individual differences in rejection sensitivity^83^, social anxiety^84^, trait self-esteem, depression^85^ and attachment style^86^ moderate the effect of social exclusion. Possibly, participants with such pre-existing (sub)clinical peculiarities in reaction to social exclusion, ostracism or labile sense of belonging might display changes in neural activity after REMS as in the present sample, but additionally report altered subjective experiences. Last, regarding the association of mental disorders and sleep disturbances^12–16^, it is possible that experimental REM-sleep suppression becomes effective on the emotional level only when applied repeatedly, in that way constituting a more realistic simulation of chronic sleep disturbances that are associated with psychiatric disorders^12–16^.

A limitation of our study is that although the use of cognitive reappraisal (CRA) strategies was effective in toning down feelings of social exclusion^87^, CRA was always applied in the second session. Therefore, we cannot rule out that habituation to the task during the second session influenced the regulation of emotionality by CRA. However, counterbalancing the view and reappraisal session would have introduced even stronger confounds, considering that participants would have most likely also engaged in CRA in the second session if they had learned about this strategy in the first session. In order to better discriminate between CRA and habituation effects one could apply a three-session-design and randomly instruct participants to use CRA either in the second or third session, as was done in an earlier study by Mauss and colleagues^46^.

A second limitation is that we exclusively investigated only one of several possible emotion regulation strategies by explicitly asking subjects to engage in CRA. CRA is generally regarded as the most efficient emotion regulation technique^88^, and as indicated by our findings, subjects are still able to utilize it even following partial sleep suppression. Possibly, the explicit instruction to engage in CRA may have elicited social desirability effects and increased the motivation to successfully engage in CRA^89^. Alternatively, one could implicitly offer subjects an opportunity for diverting their attention away from the unpleasant emotional experience in one session and thereby apply another emotion regulation technique (i.e. distraction) with less danger for social desirability effects. Future studies will need to specifically target the effect of different emotion regulation strategies and the impact that selective REMS might exert on such techniques.

Last, the suitability of the SWSS group as a control group with selective sleep suppression may be limited, since its sleep physiology was similar to the CTL group, and the suppression effect within the SWSS group was not robust, albeit higher than in the remaining groups. Future work thus needs to address this issue to more robustly compare the roles of SWS and REM sleep in emotional functioning.

Conclusively, our findings support the notion that intact REM sleep is important for next-day affective functioning^19,33,39^. However, contrary to our expectations, this statement pertains only to general negative affect and neural responses to passively experienced social exclusion, but was not evident in subjective emotional reactions. Last, REMS did not interfere with cognitive reappraisal, neither behaviorally nor neurally. It remains possible that REMS has more detrimental effects in (sub-)clinical psychiatric populations. Together, our study points to the need for further research to understand which domains of affective functioning are particularly vulnerable to REM sleep disturbances, and how this is relevant for individuals at risk for mental disorders.

## Methods

### Participants

A total of 45 participants were initially invited to take part in the experiment (29 female, age (years): *M* = 23.69, *SD* = 2.67), gave written informed consent prior to participation in the study and received financial compensation for their participation. The study protocol was approved by the institutional review board of Philipps-University Marburg in accordance with the declaration of Helsinki. All participants were recruited at Philipps-University Marburg, were fluent in German, and had normal or corrected-to-normal vision. None of them were diagnosed with neurological or psychiatric disorders (present and past), current alcohol or drug abuse, use of psychiatric medications (present and past), anatomical brain abnormalities (e.g. lesions, strokes etc.), or sleep disturbances. Due to technical issues with the polysomnographic recording in the experimental night (n = 1) or insufficient quality of the fMRI data (n = 2), 3 subjects were not included in the final analyses. The final sample thus consisted of 42 participants (27 female, age (years): *M* = 23.76, *SD* = 2.74). Participants were randomly assigned to one of three groups, which differed regarding to the sleep protocol in the experimental night (i.e. either REMS, SWSS or CTL, details below). Groups did not differ in age (CTL: *M* = 24.00 years, *SD* = 3.12; REMS: *M* = 23.06, *SD* = 2.22; SWSS: *M* = 24.60, *SD* = 2.91; *F*(2,39) = 1.09, *p* = 0.346) or gender (CTL: 8 female, 7 male; REMS: 12 female, 5 male; SWSS: 7 female, 3 male; *X*^2^(2,42) = 1.22, *p* = 0.543).

### Sleep manipulation procedure

Participants came to the laboratory for two consecutive nights—one habituation night and a subsequent experimental night (see Fig. 1). The purpose of the habituation night was to exclude sleep disorders, make participants familiar with the polysomnography (PSG) recording procedure and adapt to the environment in the sleep laboratory. The morning after the experimental night participants were taken to the fMRI facility where they completed the experimental task in the scanner, with scanning beginning between 7:00 am and 10:00 am (on average 8:16 am; see Experimental task below). During both nights PSG was recorded to monitor and identify sleep phases (see Table 1). A maximum of three participants was invited to the sleep laboratory on each night. Upon arrival on the habituation night, participants were informed about the procedure of the study and gave written informed consent. After the habituation night participants could spend the day as usual but were asked to refrain from napping, smoking and consuming stimulating foods and drinks (e.g. coffee, tea, energy drinks). On the subsequent night, participants entered a fully equipped single sleep room for PSG (Embla N7000 PSG, TNI-Medical, Würzburg, Germany) consisting of electroencephalography (EEG), electrooculogram (EOG), electromyogram of the mentalis muscle (EMG) and pulse oximetry using EMBLA N7000 (TNI-Medical). The EEG system had 9 electrodes positioned according to the 10–20 system, adhering to the guidelines of the American Academy Of Sleep Medicine (AASM)^60^, and placed on the following positions: F3, F4, C3, Cz, C4, O1, O2, M1 and M2. In case a deterioration of the PSG recordings was observed, the experimenter entered the sleep room to improve the signal by reattaching the respective electrodes. Limb movements were videotaped with an infrared camera. PSG recordings were scored by an experienced sleep technician (C.L.) at the sleep laboratory at the Department of Otorhinolaryngology at the University of Lübeck according to AASM guidelines^60^ and using Somnologica 3.3.1 (Build 1529). The technician was blinded for group assignments and hypotheses.

The experimenter turned off the lights at 10 pm and asked participants to try to fall asleep. At 6 am, lights were turned on, participants were woken up and the electrodes were removed. For those participants in the REMS and SWSS group, during the second night (i.e. the experimental night), the experimenter started an acoustic beep (80 dB, 500 Hz, 500 ms)^90^ once the polysomnographic trajectories indicated that the participant had entered the target sleep phase. After an awakening, participants were kept awake for 90 s. In case the participant did not wake up, the volume of the acoustic beep was increased, and ultimately the experimenter entered the participant’s room to turn on the lights to make sure the target sleep phase was interrupted. To control for the number and lengths of awakenings, both suppression groups were disturbed similarly often during the experimental night. In the REMS group, participants’ sleep was disturbed as soon as they entered the REM sleep stage. Awakenings for the SWSS group were selectively carried out during the non-REM sleep stage N3, defined according to AASM guidelines^60^. The control subjects were not awakened at all.

### General affect ratings

The PANAS^59^ was completed shortly before going to bed and after waking up on both nights by all participants to assess the effect of sleep manipulation on general positive and negative affect.

### Experimental task

Upon waking up after the experimental night, participants were accompanied to the functional magnetic resonance imaging (fMRI) facility. In the MRI, participants were instructed via a screen that they were about to play a ball-tossing game, i.e. the *Cyberball* task, allegedly with two other persons (see Fig. 1; a second paradigm was presented to the subjects afterwards, which involved IAPS pictures to study regulation of basic emotions, but this is not further described in the present manuscript). All three players, including the participant, were represented by avatars, i.e. green, 3D-animated stick-figures standing in a triangle on a lawn (for a detailed description of the animation see^91^). Participants were able to control the avatar on the bottom edge of the screen, while the other two avatars were placed on the left and right of the horizontal midline of the screen. The names of the participant and of the other two players were displayed next to the respective avatar. For each throw, a short sentence on the bottom of the screen indicated who threw the ball to whom. When one of the computer avatars had the ball, they waited a random amount of time between 1000 and 2000 ms before tossing the ball. When the participant had the ball, they had 2000 ms to decide where to toss the ball. In case they responded too slowly, the computer randomly selected which of the other avatars would receive the ball. The duration of each throw was 750 ms and was visualized by a series of 40 frames, showing an avatar doing a throwing movement and the red ball flying from one avatar to the catching avatar, with the latter moving its arm to catch the ball. While participants believed that the other avatars were controlled by two other anonymous persons sitting in adjacent rooms, their behavior in fact followed a predefined script (see below). Following the instructions, a short sequence of lines of text built up on the screen, making the subjects believe that the computer was being connected to a gaming server of the university on which the experiment was run. This included the request of entering an IP-address, as well as lines stating how many players were logged into the game. Subsequently, participants performed a short training session consisting of seven trials in which they received and threw the ball three times.

The task then consisted of two sessions, each of which consisted of four experimental blocks. Before each of the experimental blocks, a line of text was presented on-screen for 1500 ms telling the participant that a new round would start. In every block, a maximum number of 24 ball tosses were completed. In two blocks of every session, the participant was included in the game and repeatedly received the ball throughout the entire block (inclusion blocks, INC), having the possibility to toss the ball to one of the other avatars eight or nine times by clicking a button using the index finger (left player) or middle finger (right player). In the other two blocks (exclusion blocks, EXC), after the participant had received the ball three or four times, the paradigm was programmed so that the two avatars only tossed the ball between one another, thereby effectively excluding the participant from the game.

After having completed four blocks during which subjects simply participated in the task without additional instructions (VIEW session), a second session of the task was played. For the second session, a short on-screen text instructed participants to use cognitive reappraisal to regulate their emotions during the game (CRA session; see Fig. 1). Participants were specifically asked to reappraise the situation, in case any negative emotions should arise (“In case negative emotions arise, please try to re-evaluate the situation”). This was accompanied by the instruction to “partake in the game and try to visualize the situation as vividly as possible”, which was also presented before the first session. Subjects were asked to press the left button as soon as they were ready, and then a short break varying randomly between 1500 and 2500 ms was presented, instructing participants to wait until the other two players had indicated that they were ready. After presentation of a fixation cross for one second, the block started.

In each session, two inclusion and two exclusion blocks were presented to the subjects, with the first block of each session always being an inclusion block. In one session, inclusion and exclusion blocks were alternating, while in the other session, the initial inclusion block was followed by two consecutive exclusion blocks and a final inclusion block. The assignment of block order to the VIEW and the CRA sessions was counterbalanced across subjects. On average, EXC blocks lasted 50.93 s (*SD* = 0.62), and INC blocks lasted 45.87 s (*SD* = 2.21).

Participants were asked to rate how socially excluded they felt immediately after having completed each block of the *Cyberball*, ensuring comparability with earlier studies using this paradigm^56,92^. The low end of the scale was labelled as rejected/despised and the high end was labelled as accepted/familiar. In order to prevent that subjects could easily guess the focus of our study, and to thereby reduce social desirability effects, additional ratings of sadness, anger, and shame were obtained (sadness was described by: sad, downcast, gloomy; for anger: angry, irritated, furious, mad; for shame: abashed, embarrassed; see Supplementary Table S4 ). Each rating was displayed using a 9-point Likert-type scale ranging from 0 (not at all) to 8 (very much). Every rating was initialized at 4 (i.e. a neutral rating), and participants used presses of the right or left response buttons to move the rating to higher or lower values, respectively. The time for ratings was limited to 4 s. After each rating and the start of the next block within a session there was a short pause, jittered between 4.3 to 5.3 s. Emotion ratings (i.e., the extent to which participants felt socially excluded) for each condition (EXC, INC) and emotion regulation session (VIEW, CRA) were analyzed using repeated measures analyses of variance (rmANOVA).

### Statistical analysis

Statistical analyses were performed using JASP (Version 0.11.1^93^). Average ratings of feeling excluded for each condition (EXC, INC) and session (VIEW, CRA), as well as the percentages of the different sleep stages for the habituation and experimental nights were analyzed using analyses of variance (ANOVA). Significant interactions and main effects were followed up using paired comparisons. Since we expected that selective REMS would specifically alter affective experience and associated neural responses^22,23^, we furthermore performed an a priori planned contrast of the REMS group against both control groups, the CTL and SWSS. The alpha-level was set to 0.05, and was adjusted using Bonferroni-correction, in case multiple tests were performed.

### FMRI data acquisition and preprocessing

For each of the two experimental sessions, 130 functional volumes were recorded at 3T (Siemens Trio, Erlangen), of which the first three were discarded to allow for equilibration of T1 saturation effects. Functional volumes consisted of 36 ascending near-axial slices (voxel size = 3 × 3 × 3 mm, 10% interslice gap, FOV = 192 mm) and were recorded with TR = 2200 ms, TE = 30 ms, FA = 90°. In addition, a high-resolution anatomical T1 image was recorded consisting of 176 slices (voxel size = 1 × 1 × 1 mm, FOV = 256 mm, TR = 1900 ms, TE = 2.52 ms, 9° FA).

The MRI data were analyzed using SPM12 in Matlab 2019b. Functional MRI data were preprocessed separately for each session of the *Cyberball* paradigm. During preprocessing, images were first slice-time corrected to the middle slice and then all volumes of a given session were realigned to the first volume of that session. Subsequently, spatial normalization to MNI space was performed using unified segmentation^94^ by estimating the forward deformation fields from the mean functional image of each session and applying these to the realigned functional images. These spatially normalized images were then resliced to a voxel size of 2 × 2 × 2 mm and smoothed with an 8 mm full-width at half-maximum isotropic Gaussian kernel and high-pass filtered at 1/256 Hz.

### FMRI data analysis

The preprocessed functional images were statistically analyzed by a two-level mixed effects GLM procedure. For each participant, a statistical model was specified, including data from both experimental sessions (VIEW, CRA). For each session, the INC, EXC and rating phases were modelled as regressors of interest and the six realignment parameters estimated during spatial realignment were included to account for variance in the functional data that was due to head motion. The contrast images obtained from the individual participants were then aggregated in a random effects model on the second level to test for effects of condition and session, and to test for differences between the three experimental groups. To disentangle significant interaction effects, we ran a series of post-hoc tests on the parameter estimates extracted from the peak activation, applying Bonferroni-correction for multiple comparisons.

Psychophysiological interaction (PPI) analysis were performed separately for left and right amygdala. For each subject and each session, we extracted the first eigenvariate of activity from all voxels within an anatomical mask of the amygdala, defined using the Wake Forest University (WFU) Pickatlas (v. 2.4)^95^. Using the PPI functions provided in SPM12, the PPI terms were constructed using the eigenvariates and the contrast EXC > INC from the respective session. The eigenvariates, the contrast EXC > INC, and their PPI terms from both sessions were then taken as regressors in a first-level GLM, together with one regressor modeling the ratings phases and six realignment parameters to account for variance related to head motion. The first-level contrast images for the PPI effect were then analyzed with a second-level full factorial model, including one between-subjects factor for subject group (CTL, REMS, SWSS) and one within-subject factor for session (VIEW, CRA).

### Regions of interest (ROI) for FMRI analysis

In order to focus our analyses on the limbic system as well as the insula, regions commonly associated with affective processing^36^, we constructed an a priori mask using the WFU Pickatlas (v. 2.4)^95^. This mask comprised the union of anterior cingulate cortex, bilateral insula, bilateral hippocampus, as well as left and right amygdala and ROI analyses were performed using inference at peak-level and applying small-volume correction as implemented in SPM12.

## Supplementary information

Supplementary Information.

## Data Availability

The datasets generated during and/or analyzed during the current study are available from the corresponding author on reasonable request.
