# Supplementary material for: Selective suppression of rapid eye movement sleep increases next-day negative affect and amygdala responses to social exclusion
Source: Sci Rep. 2020 Oct 14;10:17325. doi: 10.1038/s41598-020-74169-8 (PMC7557922; doi:10.1038/s41598-020-74169-8)
Supplement: Supplementary file 1 — Supplementary Information. [file 41598_2020_74169_MOESM1_ESM.pdf]

Supplementary Information

Selective Suppression of Rapid Eye Movement Sleep Increases Next-Day Negative Affect and Amygdala Responses to Social Exclusion

Glosemeyer, R.W., Diekelmann, S., Cassel, W., Kesper, K., Koehler, U., Westermann, S., Steffen, A., Borgwardt, S., Wilhelm, I., Müller-Pinzler, L., Paulus, F.M., Krach, S., & Stolz, D.S.

Supplementary Analyses

Subjective Sleepiness

In order to assess subjective sleepiness, we aggregated two items from the Positive and Negative Affect Schedule (PANAS; [1]), namely "alert" and "active" (German: "wach", "aktiv"), in order to obtain a proxy for subjective sleepiness. We analyzed these ratings from the four time-points (evening before habituation night, morning after habituation night, evening before experimental night, morning after experimental night; see table S1 for descriptive statistics) with a 2 (within-subject factor *night*: habituation night, experimental night) by 2 (within-subject factor *time of day*: evening, morning) by 2 (between-subjects factor *REMgroup:* yes, no) ANOVA. This yielded significant main effect of *time of day* ($F(1,38)=13.21$, $p<.001$), but no significant main effects of *night* ($F(1,38)=2.95$, $p=.094$) or *REMgroup* ($F(1,38)=1.66$, $p=.206$). Further, no significant 2-way interactions (*time of day * REMgroup:* $F(1,38)=0.25$, $p=.619$; *time of day*night:* $F(1,38)=2.79$, $p=.103$; *night * REMgroup:* $F(1,38)=0.45$, $p=.508$) or 3-way interaction were found (*time of day * REMgroup * night:* $F(1,38)=2.46$, $p=.125$). When running equivalent analyses comparing the CTL group against the other two groups, the main effect of *time of day* was significant ($F(1,38)=14.43$, $p<.001$). However, as before, none of the remaining effects were significant (all *p*s>.216). Comparably, when using a between-subjects factor with three levels (CTL, REMS, SWSS), the main effect of *time of day* remained significant ($F(1,38)=14.64$, $p<.001$), but none of the remaining effects were significant (all *p*s>.098).

General Affect

Previous studies have shown that affective processing is related to low sleep quality [2] and can be impacted by unspecific sleep interruptions, i.e. not specific REM sleep or SWS interruptions [3]. To rule out the possibility that the effects in our study were simply due to such unspecific effects, we compared the change in PA and NA from the morning following the habituation night to the morning after the experimental night between the CTL and the other two groups. This analysis did not yield any significant effects (PA: $t(38)=-.77$, two-sided $p=.445$, $d=-0.25$, 95% $CI=[-0.89;0.39]$; NA: $t(38)=1.04$, two-sided $p=.305$, Cohen's $d=0.34$, 95% $CI=[-0.31;0.98]$).

Subjective Emotion Ratings

As for general affect, we additionally tested whether emotional experiences during the Cyberball differed between the CTL group and the two groups in which sleep was interrupted, and did not find any significant main effect or interaction (all *p*-values > .15).

fMRI Data

Testing for general differences in neural activity between the CTL group and the other two groups, across sessions and conditions, did not yield any significant results. Furthermore, no significant interaction effects were found with condition (EXC/INC) or with session (VIEW/CRA). Last, we tested whether the two-way interaction contrast [EXC/VIEW>INC/VIEW] > [EXC/CRA>INC/CRA] differed between the CTL group and the other groups with experimentally disturbed sleep. This analysis showed a significant 3-way interaction effect in right hippocampus (42, -12, -18; $F=29.17$, $k=8$, $p=.012$, FWE-corrected inside the a priori mask). To disentangle this interaction, we extracted the contrast estimate EXC>INC from both sessions for each participant. Testing these contrast estimates against zero, separately for groups and sessions, did not show any significant effects (all *p*-values > .089; Bonferroni-corrected, two-sided *p*-values). However, paired *t*-

tests revealed that in both groups with selective sleep suppression**,** the effect of EXC>INC in right hippocampus was larger during VIEW than during CRA (REMS: $t(16)=4.65$, two-sided $p<.001$; SWSS: $t(9)=3.54$, two-sided $p=.019$). In the CTL group this effect tended to be larger during CRA than during VIEW, but this difference did not survive Bonferroni-correction (CTL: $t(14)=-2.53$, two-sided $p=.072$; Bonferroni-corrected, two-sided *p*-values).

Psychophysiological Interaction Analysis

No significant effects were found when comparing the CTL group against the other two groups, neither in terms of a main effect, nor in terms of a significant interaction with session type.

Supplementary Tables

Supplementary Table S1. Subjective sleepiness ratings

	Habituation night				Experimental night			
	evening before		morning after		evening before		morning after	
	M	*SD*	*M*	*SD*	*M*	*SD*	*M*	*SD*
CTL	2.53	0.97	2.00	0.76	2.53	0.74	2.00	0.85
REMS	2.23	0.46	2.13	1.16	2.30	0.86	1.57	0.59
SWSS	2.70	0.68	2.15	0.58	2.45	0.80	1.85	0.78

Note. CTL = control group with regular sleep, REMS = group with selectively suppressed rapid eye movement sleep, SWSS = group with selectively suppressed slow wave sleep.

Supplementary Table S2. ANOVA of evening Positive Affect ratings (PANAS[4])

Within Subjects Effects	Sum of Squares	*df*	Mean Square	*F*	*p*
Evening	1.60	1	1.60	9.72	.004
Evening * REMSgroup	0.03	1	0.03	0.16	.688
Residual	6.24	38	0.16		
Between Subjects Effects					
REMSgroup	0.47	1	0.47	0.94	.339
Residual	19.13	38	0.50		

Note. The PANAS was scored in the evenings before the habituation night and the experimental night (factor Evening). The REMS group was compared against the other two groups (CTL, SWSS; factor REMSgroup).

Supplementary Table S3. ANOVA of evening Negative Affect ratings (PANAS[4])

Within Subjects Effects	Sum of Squares	*df*	Mean Square	*F*	*p*
Evening	0.14	1	0.14	1.99	.167
Evening * REMSgroup	0.19	1	0.19	2.68	.110
Residual	2.65	38	0.07		
Between Subjects Effects					
REMSgroup	0.02	1	0.02	0.12	.732
Residual	7.82	38	0.21		

Note. The PANAS was scored in the evenings before the habituation night and the experimental night (factor Evening). The REMS group was compared against the other two groups (CTL, SWSS; factor REMSgroup).

Supplementary Table S4. Descriptive statistics for shame, anger, sadness

	group	EXC/VIEW		INC/VIEW		EXC/CRA		INC/CRA	
		M	*SD*	*M*	*SD*	*M*	*SD*	*M*	*SD*
shame	CTL	3.43	2.38	1.63	0.79	2.10	1.66	1.20	0.41
	REMS	2.77	2.04	1.38	0.49	2.35	2.07	1.24	0.50
	SWSS	2.05	1.44	1.40	0.46	1.80	1.14	1.60	0.91
anger	CTL	4.63	2.33	1.83	0.99	2.67	1.73	1.53	0.99
	REMS	5.03	2.16	1.74	0.95	3.00	1.89	1.47	0.65
	SWSS	4.20	2.58	2.20	2.02	2.25	1.36	1.65	1.08
sadness	CTL	5.03	2.39	1.67	0.79	2.57	1.67	1.47	0.72
	REMS	3.97	2.31	1.79	0.94	3.09	2.03	1.41	0.62
	SWSS	2.60	1.71	1.90	0.88	2.05	1.04	1.90	0.97

Note. EXC = exclusion blocks, INC = inclusion blocks, VIEW = passive viewing session, CRA = cognitive reappraisal session. CTL = control group with regular sleep, REMS = group with selectively suppressed rapid eye movement sleep, SWSS = group with selectively suppressed slow wave sleep.

Supplementary Table S5. ANOVAs of Positive Affect ratings (PANAS[4]), change from evening before to morning after experimental night

		Sum of Squares	*df*	Mean Square	*F*	*p*	*η²*
ANOVA 1	group	0.17	2	0.08	0.35	.708	0.02
	Residual	8.94	37	0.24			
ANOVA 2	is REMS group	0.17	1	0.17	0.71	.404	0.02
	Residual	8.94	38	0.24			
ANOVA 3	is CTL group	0.07	1	0.07	0.31	.583	0.01
	Residual	9.04	38	0.24			

Note. Dependent variable was the difference in positive affect on the evening minus the morning of the experimental night. group = factor with 3 levels (CTL, REMS, SWSS). is REMS group = factor with 2 levels (REMS, CTL&SWSS). is CTL group = factor with 2 levels (CTL, REMS&SWSS).

Supplementary Table S6. ANOVAs of Negative Affect ratings (PANAS[4]), change from evening before to morning after experimental night

		Sum of Squares	*df*	Mean Square	*F*	*p*	*η²*
ANOVA 1	group	0.27	2	0.13	0.98	.384	0.05
	Residual	5.00	37	0.14			
ANOVA 2	is REMS group	0.17	1	0.17	1.27	.268	0.03
	Residual	5.09	38	0.13			
ANOVA 3	is CTL group	0.00	1	0.00	0.00	.999	0.00
	Residual	5.26	38	0.14			

Note. Dependent variable was the difference in negative affect on the evening minus the morning of the experimental night. group = factor with 3 levels (CTL, REMS, SWSS). is REMS group = factor with 2 levels (REMS, CTL&SWSS). is CTL group = factor with 2 levels (CTL, REMS&SWSS).

Supplementary References

[1] Watson, D., Clark, L. A. & Tellegen, A. Development and validation of brief measures of positive and negative affect: The PANAS scales. *J. Pers. Soc. Psychol.* **54**, 1063–1070 (1988).

[2] Bower, B., Bylsma, L. M., Morris, B. H. & Rottenberg, J. Poor reported sleep quality predicts low positive affect in daily life among healthy and mood-disordered persons: Sleep quality and positive affect. *J. Sleep Res.* **19**, 323–332 (2010).

[3] Finan, P. H. *et al.* Partial sleep deprivation attenuates the positive affective system: Effects across multiple measurement modalities. *Sleep* **40**, (2017).

[4] Crawford, J. R. & Henry, J. D. The Positive and Negative Affect Schedule (PANAS): Construct validity, measurement properties and normative data in a large non-clinical sample. *Br. J. Clin. Psychol.* **43**, 245–265 (2004).
